# Supplementary material for: A Single-Component Blue Light-Induced System Based on EL222 in Yarrowia lipolytica
Source: Int J Mol Sci. 2022 Jun 6;23(11):6344. doi: 10.3390/ijms23116344 (PMC9181742; doi:10.3390/ijms23116344)
Supplement: Supplementary file 1 [file ijms-23-06344-s001.zip › ijms-1699158-supplementary.pdf]

International Journal of
Molecular Sciences

MDPI

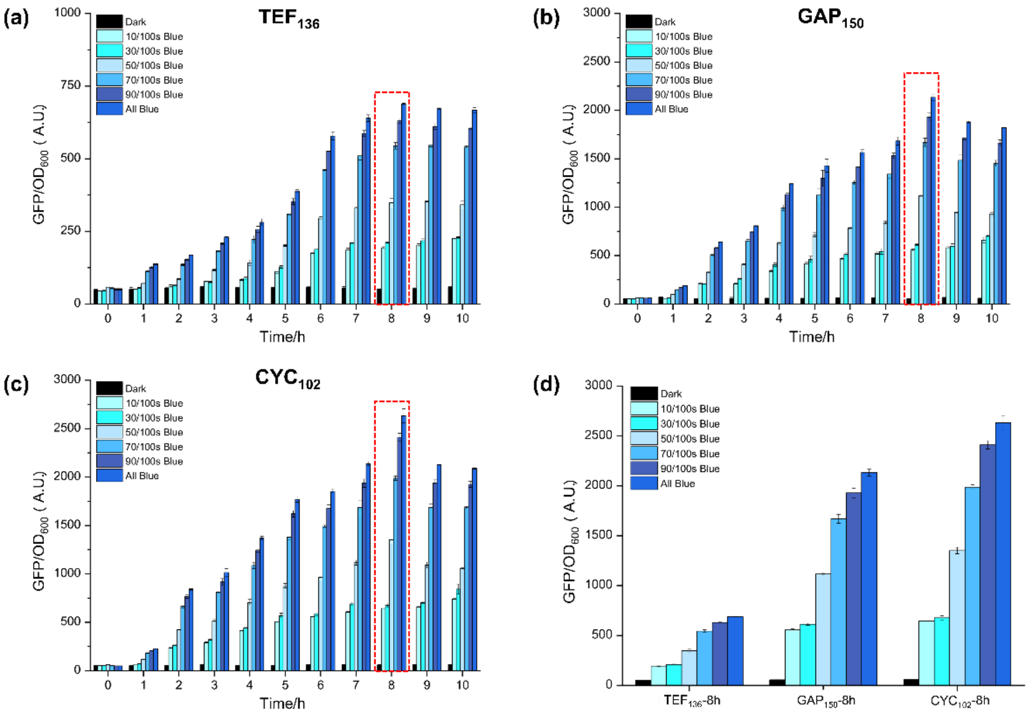

Figure S1. Responses of pYLBI systems with different core promoters to the time and dose of blue light irradiation. (a-c) Response effect of pYLBI system with different core promoter CYC_{102}, GAP_{150} or TEF_{136}. (d) Comparison of maximum induction strength of pYLBI system with different core promoters.

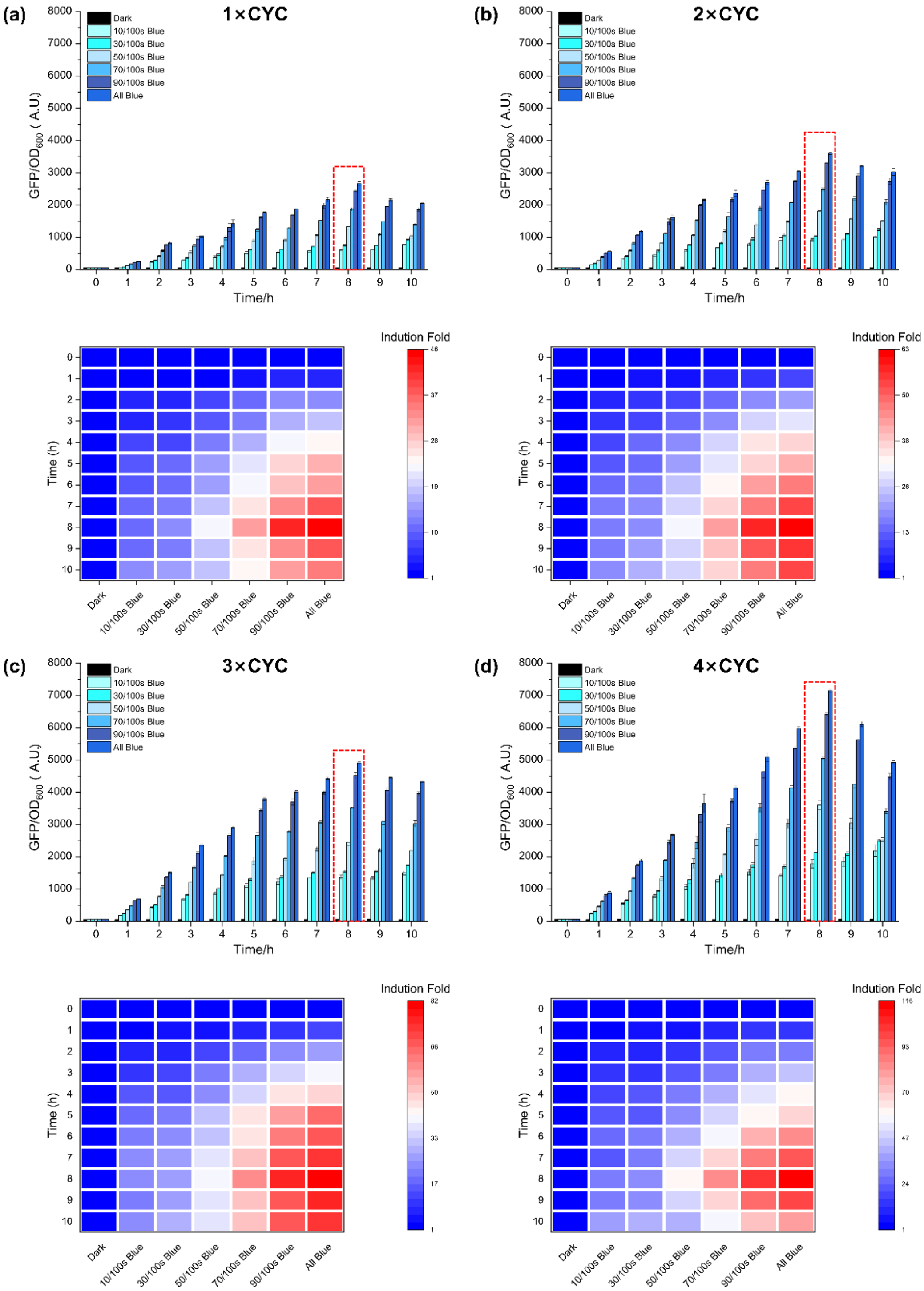

Figure S2. Responses of pYLBI systems with different copy numbers of response fragments to time and dose of blue light irradiation. (a-d) Response effect of the pYLBI system containing 1 to 4 copies of the response fragment. The red dotted line outlines the highest induction fold.

Table S1. Core/Minimal promoter sequence.

Core Promoter	Sequence
$minP_{64}$	AGACACTAGAGGGTATATAATGGAAGCTCGACTTCCAGCTTGG-CAATCCGGTACTGTTGGTAAA

TRP_{148}	GTACAATCTTGATCCGGAGCTTTTCTTTTTTTGCCGATTAA-<br>GAATTAATTCGGTCGAAAAAA-<br>GAAAAGGAGAGGGCCAAGAGGGAGGGCATTGGTGACTATTGAG<br>CACGTGAGTATACGTGATTAAGCACACAAAGGCAGCTTGGAGT
$HIS3_{188}$	CTAGTACACTCTATATTTTTTTATGCCTCGGTAATGAT-<br>TTTCATTTTTTTTTTTCCAC-<br>CTAGCGGATGACTCTTTTTTTTTCTTAGCGATTGGCATTATCACAT<br>AATGAATTATACATTATATAAAGTAATGTGATTTCTTCGAAGAA-<br>TATACTAAAAAATGAGCAGGCAAGATAAACGAAGGCAAAG
CYC_{102}	GCATGTGCTCTGTATGTATATAAAACTCTT-<br>GTTTTCTTCTTTTCTCTAAA-<br>TATTCTTTCCTTATACATTAGGACCTTTGCAGCATAAATTACTATA<br>CTTCTA
GAP_{150}	ATAATAGCGGGCGGACGCATGTCATGAGATTATTGGAAACCAC-<br>CAGAATCGAA-<br>TATAAAAGGCGAACACCTTTCCCAATTTTGGTTTCTCCTGACCCA<br>AAGACTTTAAATTTAATTTATTTGTCCCTATTTCAATCAATT-<br>GAACAACTAT
TEF_{136}	TTGTGGTTGGGACTTTAGCCAAGGGTATAAAAGACCAC-<br>CGTCCCCGAATTAC-<br>CTTTCCTCTTCTTTTCTCTCTCTCCTTGTCAACTCACACCCGAAATC<br>GTTAAGCATTTCCTTCTGAGTATAAGAATCATTCAAA
